# Application of Isothermal and Isoperibolic Calorimetry to Assess the Effect of Zinc on Hydration of Cement Blended with Slag

**DOI:** 10.3390/ma12182930

**Published:** 2019-09-10

**Authors:** Pavel Šiler, Iva Kolářová, Radoslav Novotný, Jiří Másilko, Jan Bednárek, Martin Janča, Jan Koplík, Jan Hajzler, Lukáš Matějka, Michal Marko, Přemysl Pokorný, Tomáš Opravil, František Šoukal

**Affiliations:** 1 Materials Research Centre, Faculty of Chemistry, Brno University of Technology, Purkyňova 118, CZ-61200 Brno, Czech Republic; 2 Technical University of Ostrava, Institute of Environmental Technology, 17. listopadu 2172/15, CZ-70800 Ostrava, Czech Republic; 3 Institute of Automotive Engineering, Faculty of Mechanical Engineering, Brno University of Technology, Technická 2896/2, CZ-61669 Brno, Czech Republic

**Keywords:** Portland cement, zinc, isothermal calorimetry, isoperibolic calorimetry, ground blast furnace slag

## Abstract

This work deals with the influence of zinc on cement hydration. The amount of zinc in cement has increased over recent years. This is mainly due to the utilization of solid waste and tires, which are widely used as a fuel in a rotary kiln. Zinc can also be introduced to cement through such secondary raw materials as slag, due to increased recycling of galvanized materials. The aim of this work was to determine the effect of zinc on the hydration of Portland cement, blended with ground blast furnace slag (GBFS). This effect was studied by isothermal and isoperibolic calorimetry. Both calorimetry methods are suitable for measurements during the first days of hydration. Isoperibolic calorimetry monitors the hydration process in real-life conditions, while isothermal calorimetry does so at a defined chosen temperature. Zinc was added to the cement in the form of two soluble salts, namely Zn(NO_3_)_2_, ZnCl_2_, and a poorly soluble compound, ZnO. The concentration of added zinc was chosen to be 0.05, 0.1, 0.5, and 1mass percent. The amount of GBFS replacement was 15% of cement dosage. The newly formed hydration products were identified by X-ray diffraction method (XRD).

## 1. Introduction

Portland cement is the hydraulic binding material consisting mainly of Portland cement clinker anda limited amount of gypsum [1]. Clinker phases are tricalcium silicate (abbreviation C_3_S-3CaO·SiO_2_ or Ca_3_SiO_5_, alite), dicalcium silicate (abbreviation C_2_S-2CaO·SiO_2_ or Ca_2_SiO_4_, belite), tricalcium aluminate (abbreviation C_3_A-3CaO·Al_2_O_3_ or Ca_3_Al_2_O_6_, celite) and calcium aluminoferrite (abbreviation C_4_AF-4CaO·Al_2_O_3_·Fe_2_O_3_ or Ca_4_Al_2_Fe_2_O_10_, brownmillerite) [2,3,4]. Sulfate ions from gypsum (CaSO_4_·2H_2_O) influence the reaction of C_3_A and Ca(OH)_2_ (CH, portlandite), to form ettringite(C_6_A$\bar{S}$**_3_**H_32_). Gypsum is mainly used as a cement setting time regulator [5].

Zinc can influence the hydration of cement minerals and the microstructure of hydration products [6].

The influence of zinc on clinker properties has been investigated in many works [7,8,9]. According to the literature [10], zinc is mostly bound to the interstitial phase of clinker. In the study conducted by the authors of [11], the impact of zinc on the hydration of C_3_S and C_3_A was solved. According to the results, C_3_A hydration is primarily dependent on the concentration of sulfates in cement. At the sulfate concentration higher than 2.5%, the hydration of C_3_A is slowed down [11]. Zinc also prevents the formation of Ca(OH)_2_ in the first week of C_3_A hydration. Chemical stabilization is more important than physical metal retention in the structure [12].

Zinc delays the initial hydration of C_3_S, and Zn_2_(OH)_6_·2H_2_O can precipitate [13].

Zinc also rapidly hydrolyzes and absorbs on the surface of cement grains. The zinc hydroxyl anions Zn(OH)_3_^−^ and Zn(OH)_4_^2−^ creation has been proved. Subsequently, they are transformed into insoluble CaZn_2_(OH)_6_·2H_2_O prior to the formation of portlandite.

The hydration can be suppressed until these reactions are complete, because both OH^−^ and Ca^2+^ are present in low concentrations. The formation of portlandite depends on the concentration of Ca^2+^ and OH^−^ ions in the surrounding solution afterward. It can cause a delay in the saturation of the surrounding solution. Ca(Zn(OH)_3_)_2_·2H_2_O may be formed during the induction period. This results in a delay of the saturation of pore solution and slows down the hydration reactions. The reaction of calcium heavy metal double hydroxid es on the surface of C_3_S has been described in study [13], see Equations (1)–(3):

(1) $${\mathrm{Zn}}^{2+}+2{\mathrm{OH}}^{-}\to \mathrm{Zn}{\left(\mathrm{OH}\right)}_{2}$$

(2) $$\mathrm{Zn}{\left(\mathrm{OH}\right)}_{2}+2{\mathrm{OH}}^{-}\to 2H_{2}O+{\mathrm{ZnO}}_{2}^{2-}$$

(3) $$2{\mathrm{ZnO}}_{2}^{2-}+C_{3}S/O-{\mathrm{Ca}}^{2+}+6H_{2}O\to C_{3}S/O-{\mathrm{CaZn}}_{2}{\left(\mathrm{OH}\right)}_{6}\cdot 2H_{2}O+2{\mathrm{OH}}^{-}$$

The existence of CaZn_2_(OH)_6_2H_2_O was demonstrated by Lieber [13]. Taplin furthermore studied the slow-down of the hydration [14]. Zinc increases cement penetration, probably by supporting the ettringite formation [15,16,17,18]. According to the authors of [19], zinc retards hydration reactions by poisoning CSH nucleation and/or growth.

Other concepts of zinc implementation into CSH structures and the precipitation of zinc-containing silicates have also been published [20,21,22,23,24,25].

Gineys et al. found that the element only has a small effect on the resulting strength because zinc can precipitate as hydroxides into the space between the crystals [26].

Nochaiy and his group published an article about the impact of ZnO on mechanical strength. In the first three days, a decrease in strength was recorded. After 28 days, the measured strength was higher than that of the control sample, as a result of filling by ZnO nanoparticles [27].

The effect of ZnCl_2_ and ZnSO_4_ was studied by the authors of [28] and little negative influence on the compressive strength of the samples at 28 days was observed [28].

Siler et al. used isothermal and isoperibolic calorimetry to monitor the effect of zinc on the hydration of Portland cement. They used Zn(NO_3_)_2_·6H_2_O, ZnCl_2_ and ZnO for the measurements. The degree of retardation of cement hydration caused by zinc was shown by the authors of [29].

Granulated blast-furnace slag is a common additive to cement. For this reason, it is important to monitor its behavior in combination with cement and zinc. Cement production gives rise to the emission of CO_2_ gas, which has a detrimental impact on the environment, as it is believed to play a role in accelerating global warming. The use of ground granulated blast-furnace slag (GGBFS) is one possible strategy to limit the environmental impact of Portland cement in construction [30]. GGBFS is a by-product of iron manufacture. The molten iron slag from the blast furnace is quenched with water or steam to produce a glassy and granular material, which is ground to a fine powder to produce GGBFS. The material has almost the same fineness and specific surface area as Portland cement. The material is glassy in nature and latently hydraulic, and its use in mortar and concrete has been specified by various standards. Its chemical composition can vary between different sources [31,32,33,34]. Mixing of finely ground blast furnace slag (GBFS) with cement results in physical and chemical effects. The main physical effects are: (a) the dilution effect, where more reactive cement is replaced by a less reactive component; (b) the filler effect, where the space between the grains of cement is filled by smaller particles of secondary raw materials; (c) the stimulation effect, which is the creation of new nucleation sites, typically leading to acceleration of hydration. The chemical effects are: (a) initial slag reaction, wherein the slag reacts with alkali and calcium hydroxide formed during the hydration of the cement; (b) the auto-pozzolanic reaction [35,36,37,38]. It is generally accepted that pozzolanic materials reduce early heat evolution due to the dilution effect. Pozzolan or slag incorporation is typically reported to retard and reduce the rate of hydration [36,37,38]. Some additives with pozzolanic or hydraulic properties, replacing part of the cement in the material, are proposed to reduce the hydrate conversion and, therefore, its negative effects [39]. The fixation of calcium hydroxide in the pozzolanic is usually done after a longer period of hydration but has been observed also in earlier stages of hydration [40].

This study illustrates the influence of zinc in the form of ZnO, Zn(NO_3_)_2_ and ZnCl_2_ at different concentrations on the hydration of Portland cement blended with slag. Isothermal and isoperibolic calorimeters were used to monitor the cement hydration. It is important to monitor the effect of various compounds (due to their solubility) on the hydration of Portland cement. The amount of doped salts is also important for this study. The scientific background is in showing the influence of studied compounds on the hydration of Portland cement, identifying the newly formed compounds and expressing the length of the induction period as a mathematical function. Obtained results may thus be useful for optimizing the dosage of secondary raw materials or alternative fuels in a rotary kiln.

## 2. Materials and Methods

### 2.1. Materials and Sample Preparations

The mixtures were prepared from the cement CEM I 42.5 R Mokrá–Českomoravský cement, a.s. (Heidelberg Cement, Mokrá-Horákov, Czech Republic) (x_10_ = 0.47 µm, x_50_ = 8.89 µm, x_90_ = 34.42 µm). Finely ground blast furnace slag was from Kotouč Štramberk, spol. s.r.o. (Kotouč Štramberk, spol. s.r.o., Štramberk, Czech Republic) (x_10_ = 0.78 µm, x_50_ = 5.33 µm, x_90_ = 12.97 µm). The particle size was determined by laser-diffraction method. The chemical and phase compositions of used components are shown in Table 1 and Table 2.

Zinc was added in the form of soluble compounds Zn(NO_3_)_2_∙6H_2_O, ZnCl_2_ and poorly soluble ZnO in amount of 0.05 to 1% of the cement substitution (the percentages of the replacement were always calculated to pure zinc amount in the binder). The pastes were mixed with distilled water, with the water to binder ratio equal to 0.4.

To determine the GBFS effect, a 15% cement replacement by mass was chosen.

For the measurements using an isoperibolic calorimeter, 300 g of mixture was weighed into a polystyrene crucible provided with a thermo-insulating jacket and a thermocouple. The calorimetric measurements were carried out and the calorimetric curves were obtained. For isothermal calorimetry, 7 g of mixture was dosed to a glass ampoule, which was then inserted into the instrument.

The addition of up to 5% of zinc was used for the XRD analysis because of the resolution of the X-ray diffraction apparatus itself. A 5% addition of zinc was not possible to include for calorimetric measurements, due to a significant hydration delay. The main reason for using XRD was to find new compounds in cement hydration in combination with slag and zinc. The compounds resulting from the addition of 5% were likely to be present with a lower dose of zinc, but also in a lower amount. After mixing, the samples were placed in polystyrene containers and stored in a humid environment until the time of measurement. Thereafter, they were milled in a vibratory mill and the hydration was stopped by washing in acetone. Residual acetone was removed by evaporation at 50 °C.

### 2.2. Calorimetry

In this work, isoperibolic or isothermal types of calorimeters were used. The difference between both types of calorimetry was in the conditions of measurement. Isothermal calorimetry measurements were performed under precisely defined conditions, while the isoperibolic calorimeter measures were performed under real conditions (this instrument measured at actual temperatures during hydration) [41].

The prediction and control of the rise of concrete temperature due to cement hydration is important for massive concrete structures, since large temperature gradients between the surface and the core of the structure can cause cracking and thus reduce the durability of the structure [42].

### 2.3. Isothermal Calorimetry

This method is very usable especially for the monitoring of quantity of the Portland cement hydraulic activity, pozzolanic activity and latent hydraulic properties [43,44,45,46,47,48].

Isothermal calorimetry provides continuous measurements and it enables the study of the early stage of hydration where the heat rate is relatively high. This device is well suited to longer measurement times, as opposed to the isoperibolic calorimeter [30].

The measurement of hydration heat evolution was carried out using the isothermal calorimeter TAM Air (TA Instruments, New Castle, DE, USA). The measurement was based on ASTMC1679. Quartz sand was used as a reference. The measurement was carried out at 25 °C. The sample preparation is mentioned below.

### 2.4. Isoperibolic Calorimetry

Isoperibolic calorimetry is a method based on the determination of heat released at constant ambient temperature by measuring the temperature changes. The evolved heat is obtained by subsequent numerical data integration. The required calorimeter integration constant is obtained during its calibration. The difference between isoperibolic and isothermal calorimetry is the ambient temperature of the sample. The isoperibolic method uses constant ambient temperature, while the isothermal method uses the constant temperature of the reaction system. Thus, for isothermal measurements, it is important to compensate for the temperature gradient imbalances during the temperature flow measurement by cooling or heating the reaction vials. For the study of hydration reactions, it is therefore advantageous to use a combination of both these calorimetric methods [41].

### 2.5. X-ray Diffraction Analysis (XRD)

This is a rapid analytical technique primarily used for the identification of the crystalline material phase. Analyzed material is finely ground, homogenized, and the average bulk composition is determined.

X-ray diffraction analysis was performed with the X-Ray diffractometer Empyrean (Panalytical) with Bragg–Brentano parafocusing geometry, and CuKa radiation. The measurements were done within the range from 5 to 120 °2Θ with the angular step of 0.013 °2Θ and 25s duration using automatic divergence slits to maintain constant irradiation of the sample area. The measurements were repeated four times and then summed up.

## 3. Results and Discussion

The samples were prepared outside the calorimeters. For this reason, the first peakwas not fully measured. At the very beginning of hydration, the wetting and dissolving of cement phase soccurred. The chemical reactions were mainly represented by C_3_A hydration. This peak had almost no effect on the total heat released.

In some spectra from the isothermal calorimeter, we saw that the second hydration peak was divided into two peaks, referred to as the sulfate depletion peak and the main hydration peak. This peak has been described in the literature in several ways. The formation of these peaks is associated with C_3_A hydration or ettringite transformation to monosulfate [49,50,51,52,53,54]. The transformation of part of ettringite (AFt) into calcium monosulfoaluminate (AFm) can be described by the following Equation (4) [53]:

2C_3_A + C_3_A 3CaSO_4_ 32H_2_O + 4H_2_O→3(C_3_A CaSO_4_ 12H_2_O) (4)

The effect of zinc on the hydration of pure Portland cement was studied by Siler et al [30]. A negative effect of zinc on the hydration of cement was shown in this study. The start of setting was recorded approximately after 40 h when soluble salts at the dosage of 1% zinc were used. For insoluble ZnO, the start of setting was recorded approximately after 90 h, whereas in the case of pure cement, the setting started 5 h after mixing.

### 3.1. CEMI42.5R + GBFS(RefII)

**Figure 1:**
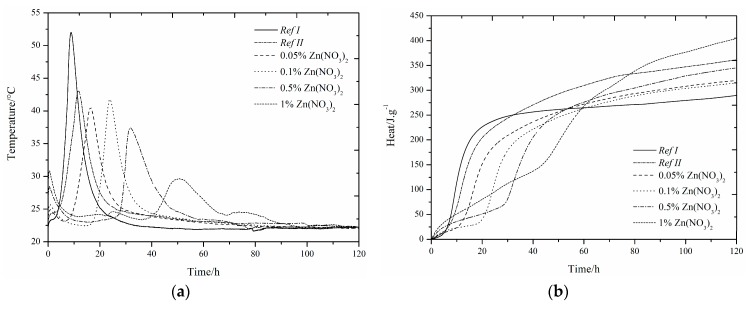
Differential (**a**) and integral (**b**) curves measured for the addition of Zn(NO_3_)_2_.

**Figure 2:**
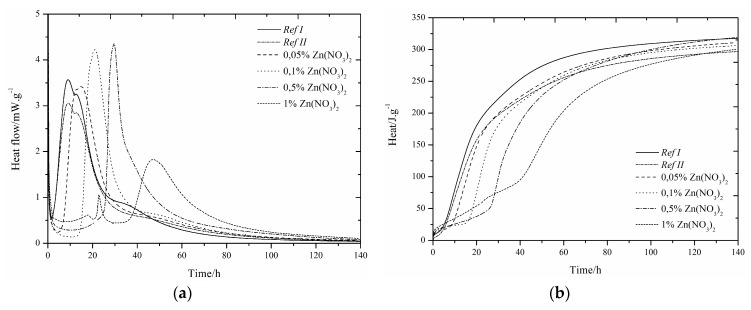
Differential (**a**) and integral (**b**) curves measured for the addition of Zn(NO_3_)_2_.

**Figure 3:**
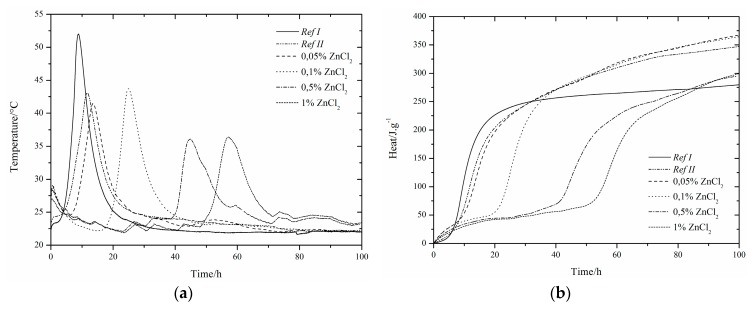
Differential (**a**) and integral (**b**) curves measured for the addition of ZnCl_2_.

**Figure 4:**
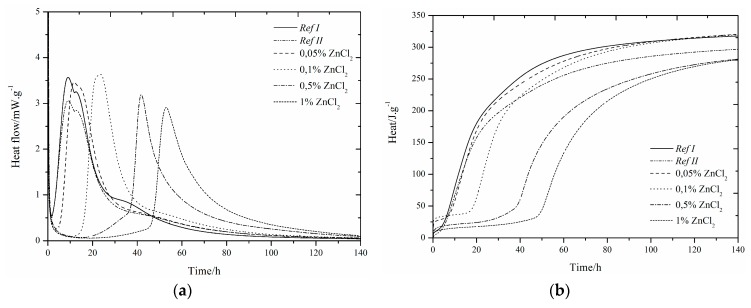
Differential (**a**) and integral (**b**) curves measured for theaddition of ZnCl_2_.

**Figure 5:**
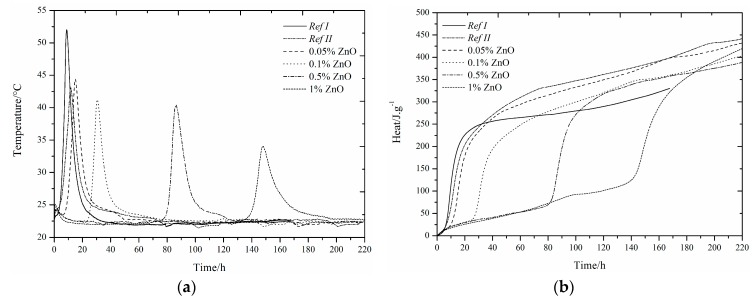
Differential (**a**) and integral (**b**) curves measured for the addition of ZnO.

**Figure 6:**
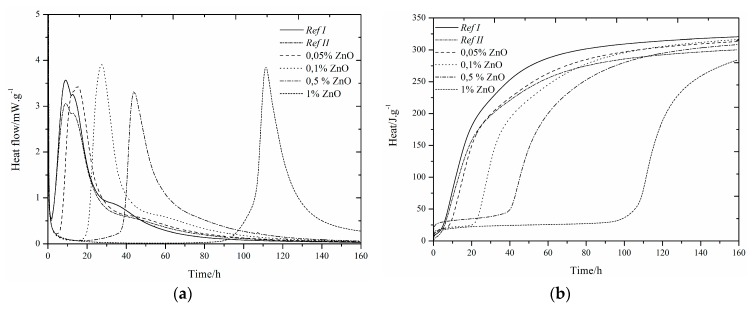
Differential (**a**) and integral (**b**) curves measured for the addition of ZnO.

The use of slag as 15% cement replacement (see Figure 1, Figure 2, Figure 3, Figure 4, Figure 5 and Figure 6) caused partial decrease in the released heat and prolongation of the induction period, due to lower reactivity of the additive compared to cement (the dilution effect, when some of the reactive material is replaced by a less reactive substance) [37]. For the same reason, the maximum heat flow was reduced in the isoperibolic measurement. The prolongation of the induction period was due to the reaction of slag with CaO. As a result, calcium ions were fixed and they were not as abundantly present as in the reaction of pure cement. But, after a certain period of time, the temperature increased, as a result of the Pozzolanic reaction [37]. Thus, the crystalline phases reacted less quickly than amorphous ones, which was manifested by a higher amount of released heat after a longer hydration time when GBFS was used. Higher reactivity of the slag over a longer period than in the case of pure cement was confirmed by a higher value of total released heat, calculated by the integration.

Several trends were found which did not depend on the zinc compound in the mixtures with slag. Zinc, in the concentration up to 0.1% did not significantly affect the maximum temperature. The samples with higher zinc content (0.5, 1%) achieved significantly lower temperature than *Ref II* (pure cement and slag). The total heat released during the isoperibolic measurement was higher for all mixtures than for the reference sample without slag and zinc (*Ref I*), due to latent reactivity of the slag, contrary to the isothermal measurements, where all measured heat flows were lower than *Ref I* due to slower slag reaction at lower temperatures.

The changes in the phase composition are shown in Table 3, Table 4, Table 5 and Table 6. The heat flow values for all samples, irrespective on the zinc content, varied up to 1 mW·g^−1^. This value could have been influenced by the formation of a nitric monosulfate analogue-3CaO·Al_2_O_3_·0.83Ca(NO_3_)_2_ 0.17Ca(OH)_2_ in mixtures with Zn(NO_3_)_2_·6H_2_O (Table 4), crystalline compounds containing chlorine-Ca_2_Al(OH)_6_Cl(H_2_O)_2_ (Table 5) as well as by the precipitation of Ca[Zn_2_(OH)_6_](H_2_O)_2_ in mixtures containing ZnO (Table 6). All of these compounds were detected after 24 h by XRD.

Increasing amounts of zinc led to more pronounced retardation of hydration reactions for non-slag samples. In the isoperibolic measurements, the delays of setting and higher values of heat released during the induction periods were noted. This was probably due to the reaction with free lime, changing the pH of the reaction environment and increasing the temperature. The free lime reaction can be supported by higher ambient temperatures, according to the achieved results, where higher released heat was measured for the slag sample *Ref II*, rather than *Ref I*. In the case of zinc samples, this heat could also have increased further.

The mixtures containing slag showed similar behavior to those without slag. Under isoperibolic conditions, the first peak was increased due to exothermic reactions [29].

Total released heat decreased with increasing zinc, content due to the slow-down of hydration. During the hydration, leachable compounds of zinc were also formed [29]. Higher heat flow than for *Ref I* and *Ref II* was measured during isothermal measurements for all compounds with 0.1% Zn. For isoperibolic measurements, the highest achieved value was recorded for *Ref I*.

### 3.2. CEMI + GBFS + Zn(NO_3_)_2_∙6H_2_O

#### 3.2.1. Isoperibolic Calorimetry

The same small exothermic peak around 25 h, as in the samples without the addition of slag, was found when the sample with 1% Zn was measured under isoperibolic conditions [13,14,29,54]. For the sample with 1% of zinc, a lower maximum temperature was reached, the reaction rate was also reduced and the peak was lower and wider. A decrease in temperature was observed for *Ref II* compared to *Ref I*. This can be explained by the presence of less reactive slag particles. With increasing zinc concentration, the maximum temperature dropped, unlike for the pure cement samples, where the 0.1% zinc concentration increased the temperature of the sample over that of *Ref I*. The temperature reduction can be explained by the larger amount of zinc incorporated into amorphous structures. After 24 h, the nitric oxide monosulfate analogue 3CaO·Al_2_O_3_·0.83Ca(NO_3_)_2_·0.17Ca(OH)_2_wasdetected by X-ray diffraction (Table 4).The heat released after 100 h was higher than for *Ref I* in all samples, and for the sample with 1% Zn in the form of Zn(NO_3_)_2_, it was even higher than for *Ref II*. This effect was due to higher slag reactivity in later hydration times, which can react very quickly because of its high amorphous phase content. These data demonstrate the effect of zinc at low concentrations as a supporter of slag reactions. Higher content of doped Zn(NO_3_)_2_·6H_2_O may also affect the pH of the hydration reaction in relation to the undetectable amount of portlandite present (measured by XRD). The increase of evolved heat is due to the exothermic dissolution of increasing amounts of Zn(NO_3_)_2_∙6H_2_O and the formation of nitric analogue of monosulfate 3CaO∙Al_2_O_3_∙0.83Ca(NO_3_)_2_∙0.17Ca(OH)_2_.

#### 3.2.2. Isothermal Calorimetry

Slag samples showed similar behavior to those without slag [29]. As described above, at higher zinc concentrations (1%) we observed the fusion of the main hydration peak and sulfate depletion peak [29,49,50,51,52,53,54]. Another peak was also recorded around 22 h by isoperibolic calorimetry, as a result of the dissolution of Zn(NO_3_)_2_ or the formation of new compounds [13,14].

Furthermore, the peak related to the conversion of ettringite to monosulfate [50,51] for the specimen with low zinc concentration was shifted from between 30 and 40 h to between 40 and 60 h and was not as visible as for samples without slag.

Thanks to high inhibiting action of zinc, the biggest influence of zinc was for samples with 1%. There is a possibility of the highest changes (pH value, temperature, evolved heat, etc.) occurring, and therefore the biggest influence taking place, because of the Zn^2+^ and NO_3_^−^ ions themselves [55]. In view of retardation time, the results from isothermal and isoperibolic calorimetry are comparable.

The XRD analysis shows the nitric analogue of monosulfate-3CaOAl_2_O_3_∙0.83Ca(NO_3_)_2_∙0.17Ca(OH)_2_ (Table 4) already after 24 h, unlike the samples with pure cement, where it was detected after 7 days. This compound may cause an increase in heat flow. For the mixtures with zinc content of 1%, it is possible to see the suppression of the development of heat flow. It is likely that the formation of this compound proceeds before the second major peak appears. This corresponds to the peak after 20 h of hydration as well as for pure cement samples. The highest heat that evolved during the 140 h of hydration was recorded for the *Ref I* sample. This difference was not very large, compared to *Ref II*, but it does show a different behavior of slag specimens when measured at constant temperature and under real conditions.

### 3.3. CEMI + ZnCl_2_

#### 3.3.1. Isoperibolic Calorimetry

A small peak between 20 and 30 h was found. Measured curves were quite similar to the curves for Zn(NO_3_)_2_. There was almost no acceleration effect of ZnCl_2_ as observed also in high dosage. Thus, it can be problematic to eliminate the negative effect of zinc through chloride hydration accelerators. As a result of the presence of slag, all measured maximum temperatures were noticeably lower than for *Ref I*. The crystalline zinc compounds Zn_5_(OH)_8_Cl_2_H_2_O and Ca_2_Al(OH)_6_Cl(H_2_O)_2_ were detected after 24 h (Table 5). The amount of Zn_5_(OH)_8_Cl_2_H_2_O increased over the 7 days of hydration, while the amount of Ca_2_Al(OH)_6_Cl(H_2_O)_2_ was constant and any present amount was created within one day. The presence of Ca_2_Al(OH)_6_Cl(H_2_O)_2_ is important for the behavior of zinc as a hydration protective membrane, which slows the hydration [29]. The temperature decreases for the most concentrated samples, due to a higher amount of zinc bound to the amorphous phase, or because free zinc ions may be present in the cement paste due to the lack of suitable sites for incorporation into the hydration grain membrane. This option corresponds to the easier release of Zn^2+^ ions during leaching after 24 h.

For soluble compounds Zn(NO_3_)_2_ and ZnCl_2_, two identical trends were found. As the amount of zinc increased, the induction period increased and the maximum temperature reached from the concentration of 0.5% was reduced. The presence of different NO_3_^−^ and Cl^−^ anions affected the behavior only slightly.

These results are in accordance with the behavior of samples without slag, published in study [29].

For all samples, higher total evolved heat values after 100 h were recorded, in comparison to *Ref I*, which explains the presence of slag that was more reactive at later stages of hydration. Furthermore, the pH was affected, as the X-ray showed no precipitation of portlandite. The mixtures with 0.05 and 0.1% of zinc showed a higher value of total released heat than *Ref II*. In contrast, the samples with the concentration of zinc of 0.5 and 1% showed a lower amount of heat than *Ref II*. The differences between the zinc mixtures can be due to imperfect homogeneity of the sample. Higher amounts of released heat can be also explained by the change of the environment (pH or ionic strength of the solution) due to ZnCl_2_, which then helps slag react.

In addition, Zn_5_(OH)_8_Cl_2_H_2_O was formed immediately within 24 h in the presence of ZnCl_2_ in the mixtures, whereas in the case of the Zn(NO_3_)_2_·6H_2_O samples, all zinc was present in the amorphous phase or in the solution. The question remains, though, regarding the pure effect of the anions of respective salts, which also form crystalline compounds 3CaOAl_2_O_3_∙0.83Ca(NO_3_)_2_∙0.17Ca(OH)_2_ and Ca_2_Al(OH)_6_Cl(H2O)_2_ after 1 day.

For bothanions, the measured maximum temperatures achieved were similar for different concentrations of zinc at low concentrations (up to 0.1%). Higher concentrations showed a more pronounced retardation effect of chloride anions, unlike pure cement [29], where 1% of zinc in the form of chlorides acted as a hydration accelerator. This fact may indicate a problem with lower efficiency of accelerators when using Portland cement mixed with slag. The measured maximum temperatures for lower concentrations were similar, but for 1% of Zn in the form of ZnCl_2_, the same maximum temperature as for the sample with 0.5% was measured, in contrast to Zn(NO_3_)_2_, where the temperature was significantly reduced. When comparing the total released heat, a more pronounced retardation effect of ZnCl_2_ was evident.

#### 3.3.2. Isothermal Calorimetry

Due to doping the samples with zinc, the main hydration peak gradually increased and then merged, as well as the samples with Zn(NO_3_)_2_ and in the case of pure cement. The course of hydration was similar for both soluble compounds, but with ZnCl_2_ the hydration slowed a little more.

Also, from these measurements it can be observed that ZnCl_2_ slows down the hydration more than Zn(NO_3_)_2_. The heat flow also decreases. These differences may be due to the formation of crystalline compounds.

For the samples with a small amount of zinc in the mixture (0.05 and 0.01%), total evolved heat was slightly higher than for *Ref I*, but for a higher amount of zinc (0.5 and 1%), less heat than for *Ref II* was measured. The increase of heat can be attributed to the precipitation of Zn_5_(OH)_8_Cl_2_H_2_O and Ca_2_Al(OH)_6_Cl(H_2_O)_2_ (Table 5). Chlorine reactions can occur together with zinc in a cement paste environment to form Zn_5_(OH)_8_Cl_2_H_2_O, and zinc can react with amorphous compounds. When zinc is depleted, chlorine can react to form a compound where zinc is replaced with calcium and aluminum, which again leads to the consumption of calcium ions [29].

The comparison of the influence of soluble salts on the hydration measured by an isothermal calorimeter can be summarized in the following points. As in the case of Zn(NO_3_)_2_·6H_2_O samples, the concentration up to 0.1% of ZnCl_2_ also led to a gradual increase of the first peak above the second peak. For the samples with concentration of zinc of 0.5% no difference between these two peaks was found. Due to the zinc inhibition, the second peak was delayed and the reactions occurred within the same time period. The heat flow increased in both test compounds up to the concentration of zinc of 0.1%.Samples with the highest amount of zinc (1%) had the lowest heat flow values. The difference was observed for the mixtures with 0.5% of zinc in the presence of Zn(NO_3_)_2_·6H_2_O. There was still an increase in heat flow in contrast to a visible decrease for ZnCl_2_. The reduction may be due to the depletion of places suitable for Zn^2+^ ions and their incorporation into Zn_5_(OH)_8_Cl_2_H_2_O, as opposed to mixtures with Zn(NO_3_)_2_·6H_2_O, where the precipitation of Ca[Zn_2_(OH)_6_](H_2_O) (Table 4) was observed after a long time. Total released heat of samples with Zn(NO_3_)_2_·6H_2_O was higher than for *Ref II*, independent of zinc concentration, unlike the mixtures with ZnCl_2_ where less released heat than for *Ref II* was measured in the most concentrated samples (0.5 and 1%). This decrease may be due to the inhomogeneity of the sample, as previously described. Zinc in the form of Zn(NO_3_)_2_·6H_2_Oat and concentration of 0.05% prolonged the induction period by 1.2 h when compared to the equally doped ZnCl_2_ sample. With a further increasing amount of zinc, a more pronounced retardation effect on ZnCl_2_ mixtures became evident. The evolved heats during the induction periods were lower for samples with Zn(NO_3_)_2_·6H_2_O, except for the zinc concentrations of 0.5 and 1%. For both compounds, increasing zinc concentrations increased the heat released during the induction periods. The influence of anions of the selected zinc compounds alone is not so important, but cannot be neglected.

### 3.4. CEMI + ZnO

#### 3.4.1. Isoperibolic Calorimetry

The highest degree of retardation occurred when Zn Owas addedto pure cement samples [29].

In comparison with admixture-free samples, higher amounts of ZnO are present in slag mixtures during the hydration, which indicates slower reaction by the addition of slag. Due to slag, the number of calcium ions is reduced, but an alkaline environment is important for the dissolution of ZnO. Therefore, it starts to react only after a certain period of time, which has the most significant impact on mechanical strengths after 24 h.

The heat released during the hydration was calculated after 220 h because of the longer time required for the hydration reaction in the presence of ZnO. For all samples, more heat than for *Ref I*, but less than for *Ref II*, was released. There is a reduction in evolved heat in the presence of zinc. The cement paste environment is affected at a later stage, when the slag should react more intensively to increase the heat. Already after 24 h, the Ca(Zn_2_(OH)_6_](H_2_O)_2_compoundwasdetected, which was also the case in pure cement samples, but in a relatively high proportion of 5% of zinc (Table 6).

The differences in maximal reached temperatures were small. The exception was only for the case of the highest addition of zinc. ZnO in 1% significantly slowed down the hydration, mainly due to its low solubility. The zinc dissolution may have taken longer than the given measurement time. A slight decrease in temperature could also have been caused by the formation of crystalline compound Ca[Zn_2_(OH)_6_](H_2_O)_2_ or by the incorporation of zinc into amorphous compounds.

ZnO showed the highest rate of hydration retardation of all compounds tested. There were no other significant peaks found in the calorimetric curve due to long ZnO dissolution time.

Comparing the isoperibolic measurements of all test compounds, the following differences were found. The maximum sample temperature with ZnO was comparable to *Ref II* unlike the samples containing Zn(NO_3_)_2_·6H_2_O and ZnCl_2_with the concentration of zinc of 0.5%. This was due to the very poorly soluble nature of ZnO. As for soluble salts, the total heat released was higher than for *Ref I*. Released heat decreased with an increasing amount of zinc. All monitored concentrations reached lower hydration temperatures than *Ref II*, except for the lowest concentration (0.05%), where a small difference may have been due to the inhomogeneity of the sample. The increase of evolved heat above the value of the sample without slag and zinc was determined by the slag reaction itself, which was more reactive in later stages of hydration due to its high amorphous phase content.

The reduction of heat in the zinc mixtures could have been due to its presence in the amorphous phase. Increasing concentrations of zinc also increased the heat released during the induction periods, which was also the case of soluble salts. The increase of this heat was caused by partial dissolution of compounds, but also by the precipitation of other newly formed compounds with zinc after 24h Ca[Zn_2_(OH)_6_](H_2_O)_2_ (Table 6) and Zn_5_(OH)_8_Cl_2_H_2_O (Table 5) and anions of soluble salts 3CaO·Al_2_O_3_·0.83Ca(NO_3_)_2_·0.17Ca(OH)_2_·9.5H_2_O (Table 4) and Ca_2_Al(OH)_6_Cl(H_2_O)_2_ (Table 5). As for pure cement samples, with ZnO addition, the most marked retardation of hydration was measured independent of zinc concentration. This was also due to slower dissolution of the compound.

#### 3.4.2. Isothermal Calorimetry

The isothermal calorimetry curves with ZnO addition were very similar for soluble compounds up to 0.1% concentration. This behavior was different from real conditions of isoperibolic calorimetry, where large differences between soluble and insoluble compounds were measured.

The sulfate depletion peak was found only for the amount of zinc of 0.05%. The concentration of zinc up to 0.5% increased the heat flow above *Ref II*. A lower value was measured for the sample with 1% Zn, due to significant prolongation of hydration, but it could be assumed that this sample would increase over *Ref II* due to later reactions. All measured samples showed lower values than *Ref I*. An increase in total released heat over *Ref I* was not observed, unlike the samples without the addition of slag.

The measured values of maximum heat flow were similar to those of pure cement without slag [29]. The highest heat flow value was measured for the sample with the highest ZnO content.

This effect was different from the soluble compounds, due to high ZnO retardation. Due to higher hydration time, more binder was able to react. No small peak was also found on the hydration curve.A double rate of retardation was observed for soluble compounds.

### 3.5. The Comparison of Induction Period Length

**Figure 7:**
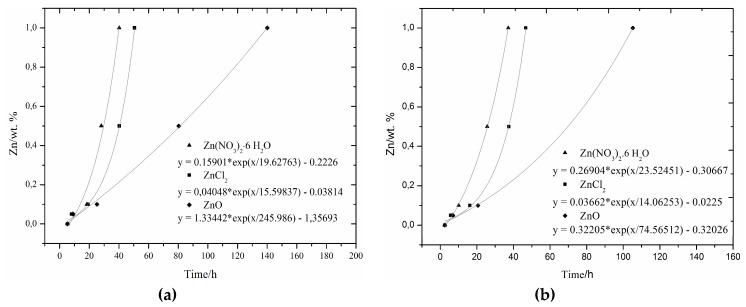
Comparison of the lengths of induction periods for isoperibolic (**a**) and isothermal (**b**) calorimetry.

The lengths of induction periods were interleaved to quantify the effect of zinc by the same exponential dependence, as in the case of study [29]. The data are displayed in Figure 7. The exponential dependence used can be expressed by Equation (5):

(5) $$y=A_{1}\cdot exp\left(\frac{-x}{t_{1}}\right)+y_{0}$$

Since the *t*_1_ value was negative for all samples, we can rewrite this to Equation (6):

(6) $$y=A_{1}\cdot exp\left(\frac{x}{t_{1}}\right)+y_{0}$$

When comparing obtained data, a very good match of the experimental data with the regression curve was found, as in the case of pure cement samples.

The measured values of the lengths of induction periods for Zn(NO_3_)_2_ and ZnCl_2_ are almost identical for isoperibolic and isothermal calorimetry measurements. In the isoperibolic calorimetry, higher degree of retardation under real conditions can be seen with the addition of ZnO. As for the samples without the addition of secondary raw materials, the hydration was most prolonged by very poorly soluble ZnO and least by Zn(NO_3_)_2_.

In this article, a 15% cement replacement by slag was used. Using a higher amount of slag will increase hydration time [37]. The retarding effect of slag with increasing amounts is not so pronounced in pure cement. When slag and zinc are combined, a more significant retardation effect can be expected, with increasing slag amount due to the modification of the gel membrane [7,8,9,10,11,12,13]. Slag does not react as quickly as cement in the early stages of hydration, which results in lower maximum temperature; however, in the later stages of hydration, the pozzolans reaction especially produces more heat than the reactions of other components [37]. For this reason, the maximum temperature (for isoperibolic measurement) will be reduced as the amount of slag increases. This reduction is likely to be more pronounced when combining zinc with cement. There will probably not be such large changes under isothermal conditions and maximum heat flow values will be comparable for different zinc additions. As a result of the lower reactivity of the slag, the evolved heat especially in the first hours of hydration will decrease and the zinc retardation rate would be more noticeable. After a few hours (possibly days), there would be higher heat generation and the total heat released would be higher than the heat measured for a sample containing only cement [37]. Isothermal conditions are not so advantageous for a faster pozzolanic reaction. For this reason, there is likely to be a further reduction in heat with a higher addition of slag and a more significant retardation caused by zinc will also be noticeable.

The aim of this paper is primarily to show both the negative effect of zinc as a hydration retarder and also to show the difference between the results of two types of calorimetry. The most significant effect of the measurement method was shown especially in the case of ZnCl_2_ [29] in combination with pure cement, where at a dose of 1%, the hydration was slightly accelerated, as compared to the dose of 0.5%. This effect was not observed in the slag sample. For other additives, acceleration of hydration can be expected due to the presence of Cl^−^ or NO_3_^−^ ions. This acceleration will be particularly visible in isoperibolic measurements. The influence of these ions on the acceleration course must always be investigated for each additive separately. The results measured with isoperibolic calorimeter also show a higher total evolved heat value than the reference samples due to the pozzolanic reaction course. This effect is not apparent in isothermal calorimetry results due to different conditions which are not so advantageous for rapid course of pozzolanic reaction.

### 3.6. XRD Results

The XRD method was used for the identification of phase composition. This identification is important for better understanding the mechanism of the zinc effect on cement hydration. The evaluation from XRD analysis was made using the Rietveld Analysis of XRD Patterns. The results are shown in Table 3, Table 4, Table 5 and Table 6.

## 4. Conclusions

This work focused on the monitoring of influence of zinc on the hydration of Portland cement blended with 15% slag using isoperibolic and isothermal calorimetry. Zinc was doped in the form of two soluble salts Zn(NO_3_)_2_·6H_2_O, ZnCl_2_ and ZnO which is very poorly soluble.

The usage of slag as 15% cement replacement caused a partial decrease in released heat and a prolongation of the induction period due to lower reactivity of the additive compared to cement (the dilution effect, when a part of reactive material is replaced by less reactive substance). This fact showed a synergic effect of the combination of zinc and slag on the prolongation of hydration. The prolongation of induction period was due to the reaction of slag with CaO. After a certain period of time, the temperature increased, as a result of the pozzolanic reaction. Higher reactivity of slag over a longer period than in the case of pure cement was confirmed by the higher value of released heat calculated by integration.

Increasing amounts of zinc led to more pronounced retardation of hydration reactions for samples without slag. Zinc up to the concentration of 0.1% did not affect the maximum temperature significantly. The samples with higher zinc content (0.5, 1%) achieved significantly lower values than the reference sample with pure cement and slag. The total released heat was higher than that which was measured for the samples without slag, as a result of the latent reactivity of slag. Samples measured with isoperibolic calorimeters achieved higher values of total evolved heat than the reference samples, while for isothermal calorimeters the highest value was recorded for pure cement.

Low exothermic peak was found in the mixtures with soluble compounds between 20 and 30 hour, as well as in the samples without the addition of slag. For higher concentrations of ZnCl_2_, a more pronounced retardation effect, due to the presence of chloride anions, was manifested in contrast to pure cement, where the use of ZnCl_2_ led to the action of chloride ions as hydration accelerators.

The addition of ZnO led to the most significant hydration prolongation, independent of the zinc concentration (1% of the addition up to 220 h). This period was longer than was measured for pure cement samples. This is due to slower dissolution of ZnO in the presence of slag.

The lengths of the induction periods were interleaved to quantify the effect of zinc on the exponential dependence. Very good match of the experimental data with the regression curve was found.

## Figures and Tables

**Table 1:** Chemical compositions of used components as % by weight.

Component	Cement %	GBFS %
CaO	65.11	43.43
SiO_2_	18.90	38.83
Al_2_O_3_	4.23	7.26
Fe_2_O_3_	3.38	-
MgO	1.02	8.49
SO_3_	3.04	-
S^2−^	0.04	-
Cl^−^	0.05	-
K_2_O	0.75	0.49
Na_2_O	0.15	0.40
ZnO	0.00005	-
LOI	3.06	1.10

**Table 2:** Phase compositions o fused components.

Component	Cement %	GBFS %
C_3_S	69.83	N/A
C_2_S	11.23	N/A
C_3_A	6.89	N/A
C_4_AF	11.20	N/A
Amorphous phase	N/A	87.27
Calcite	N/A	3.07
Mervinite	N/A	6.75
Gehlenite	N/A	3.01

**Table 3:** Mineral composition of reference sample with cement and ground blast furnace slag (GBFS).

Hydration [day]	Ca_3_SiO_5_ Hatrurite (alite)	Ca_2_(SiO_4_) Larnite (belite)	Ca_2_(Fe_2_O_5_) Brownmillerite	$C_{6}A{\bar{S}}_{3}$H_32_ Ettringite	Ca(OH)_2_ Portlandite	Ca(CO_3_) Calcite	(Mg_0.67_Al_0.33_(OH)_2_) (CO_3_)_0.165_(H_2_O)_0.48_ Hydrotalcite	(Mg_6_Fe_2_(OH)_16_ (CO_3_)(H_2_O)_4_)_0.25_ Sjogrenite
**1**	11.1	41.9	8.2	5.0	24.1	10.2	-	-
**7**	9.2	28.4	5.3	4.2	38.1	14.3	2.4	-
**28**	8.1	27.8	5.2	5.9	39.2	12.5	3.5	-
**90**	9.3	18.0	5.2	7.1	41.7	13.1	5.0	1.1

**Table 4:** Mineral composition of sample with Zn(NO_3_)_2_∙6H_2_O.

Hydration [day]	Ca_3_SiO_5_ Hatrurite (alite)	Ca_2_(SiO_4_) Larnite (belite)	Ca_2_(Fe_2_O_5_) Brownmillerite	$C_{6}A{\bar{S}}_{3}H_{32}$ Ettringite	Ca[Zn_2_(OH)_6_] (H_2_O)_2_	CaCO_3_ Calcite	3CaO∙Al_2_O_3_∙0.83Ca(NO_3_)_2_∙0.17Ca(OH)_2_∙9.5H_2_O	SiO_2_	Ca_4_Al_2_O_6_ (CO_3_)_0.67_ (SO_4_)_0.33_ 11H_2_O
**1**	12.1	52.8	4.1	11.2	-	13.3	4.1	1.1	-
**7**	12.2	55.2	4.2	10.5	-	15.2	6.3	-	-
**28**	13.1	52.7	4.0	11.4	-	13.4	7.1	-	-
**90**	14.3	50.3	3.3	17.0	2.3	13.2	5.4	-	2.3

**Table 5:** Mineral composition of sample with ZnCl_2_.

Hydration [day]	Ca_3_SiO_5_ Hatrurite (alite)	Ca_2_(SiO_4_) Larnite (belite)	FeAlO_3_(CaO)_2_ Brownmillerite	$C_{6}A{\bar{S}}_{3}$H_32_ Ettringite	Ca(CO_3_) Calcite	Ca[Zn_2_(OH)_6_] (H_2_O)_2_	Ca_2_Al(OH)_6_Cl (H_2_O)_2_ Hydrocalumite	Zn_5_(OH)_8_Cl_2_H_2_O Siminkolleite
**1**	14.3	59.5	4.3	8.2	10.0	-	3.6	1.0
**7**	12.4	60.9	4.2	8.6	8.3	-	6.2	2.3
**28**	12.1	60.8	3.3	7.5	9.0	-	6.4	2.3
**90**	13.2	55.3	2.4	10.0	9.1	5.2	7.1	1.2

**Table 6:** Mineral composition of sample with ZnO.

Hydration [day]	Ca_3_SiO_5_ Hatrurite (alite)	Ca_2_(SiO_4_) Larnite (belite)	FeAlO_3_ (CaO)_2_ Brown Millerite	$C_{6}A{\bar{S}}_{3}H_{32}$ Ettringite	Ca(OH)_2_ Portlandite	Ca[Zn_2_(OH)_6_] (H_2_O)_2_	ZnO	CaCO_3_ Calcite	CaSO_4_ 2H_2_O Gypsum	Ca_8_Al_2_ Fe_2_O_12_CO_3_(OH)_2_ 22H_2_O	Ca(SO_4_) (H_2_O)_2_	CaCO_3_(H_2_O) Monohydrocalcite	Ca_4_Al_2_O_6_ (CO_3_)_0.67_ (SO_4_)_0.33_∙11H_2_O
**1**	14.3	54.2	5.0	4.3	-	5.2	6.3	9.2	1.0	1.3	-	-	-
**7**	14.2	56.8	4.3	4.4	-	4.3	5.2	8.3	-	-	1.2	-	-
**28**	12.5	43.6	4.2	6.2	-	4.2	2.1	8.5	-	6.9	-	7.5	-
**90**	15.0	36.8	6.5	9.5	16.3	3.5	-	12.6	-	-	-	-	3.6

## References

[1] Glasser F.P., Marchand J., Samson E. (2008). Durability of concrete—Degradation phenomena involving detrimental chemical reactions. Cem. Concr. Res..

[2] Lawrence C.D., Hewlett P.C. (2004). The production of low-energy cements. Lea’s Chemistry of Cement and Concrete.

[3] Palou M.T., Šoukal F., Boháč M., Šiler P., Ifka T., Živica V. (2014). Performance of G-Oil Well cement exposed to elevated hydrothermal curing conditions. J. Therm. Anal. Calorim..

[4] Dweck J., Melchert M.B.M., Cartledge F.K., Leonardo R.S., Filho R.D.T. (2016). A comparative study of hydration kinetics of different cements by thermogravimetry on calcined mass basis. J. Therm. Anal. Calorim..

[5] Li C., Lu X., Jing G., Ye Z., Wang S., Cheng X. (2018). The effect of gypsum on the hydration of alite–belite–ferrite phase system. J. Therm. Anal. Calorim..

[6] Gawlicki M., Czamarska D. (1992). Effect of ZnO on the hydration of Portland cement. J. Therm. Anal. Calorim..

[7] Gineys N., Aouad G., Damidot D. (2010). Managing trace elements in Portland cement—Part I: Interactions between cement paste and heavy metals added during mixing as soluble salts. Cem. Concr. Compos..

[8] Gineys N., Aouad G., Damidot D. (2011). Managing trace elements in Portland cement—Part II: Comparison of two methods to incorporate Zn in cement. Cement and concrete composites. Cem. Concr. Compos..

[9] Murat M., Sorrentino F. (1996). Effect of large additions of Cd, Pb, Cr, Zn to cement raw metal on the composition and the properties of the clinker and cement. Cem. Concr. Res..

[10] Andrade F.R.D., Maringolo V., Kihara Y. (2003). Incorporation of V, Zn and Pb into the crystalline phases of Portland clinker. Cem. Concr. Res..

[11] Olmo I.F., Chacon E., Irabien A. (2001). Influence of lead, zinc, iron (III) and chromium (III) oxides the setting time and strength development of Portland cement. Cem. Concr. Res..

[12] Barbir D. (2017). Effects of Mud from a Zinc-plating Plant and Zeolite Saturated with Zinc on Portland Cement Hydration and Properties of Hardened Cement Pastes. Chem. Biochem. Eng. Q..

[13] Chen Q.Y., Tyrer M., Hills C.D., Yang X.M., Carey P. (2009). Immobilisation of heavy metal in cement-based solidification/stabilisation: A review. Waste Manag..

[14] Weeks C., Hand R.J., Sharp J.H. (2008). Retardation of cement hydration caused by heavy metals present in ISF slag used as aggregate. Cem. Concr. Compos..

[15] Trussell S., Spence R.D. (1994). A review of solidification/stabilization interferences. Waste Manag..

[16] Asavapisit S., Fowler G., Cheeseman C.R. (1997). Solution chemistry during cement hydration in the presence of metal hydroxide wastes. Cem. Concr. Res..

[17] Hamilton I.W., Sammes N.M. (1999). Encapsulation of steel foundry bag house dusts in cement mortar. Cem. Concr. Res..

[18] Qian G.R., Shiy J., Cao Y.L., Xu Y.F., Chui P.C. (2007). Properties of MSW fly ash–calcium sulfoaluminate cement matrix and stabilization/solidification on heavy metals. J. Hazard. Mater..

[19] Ataie F.F., Juenger M.C.G., Taylor-Lange S.C., Riding K.A. (2015). Comparison of the retarding mechanisms of zinc oxide and sucrose on cement hydration and interactions with supplementary cementitious materials. Cem. Concr. Res..

[20] Ziegler F., Scheidegger A.M., Johnson C.A., Dähn R., Wieland E. (2001). Sorption Mechanisms of Zinc to Calcium Silicate Hydrate: X-ray absorption fine structure (XAFS) investigation. Environ. Sci. Technol..

[21] Rose J., Moulin I., Masion A., Bertsch P.M., Wiesner M.R., Bottero J.-Y., Mosnier F., Haehnel C. (2001). X-ray Absorption Spectroscopy Study of Immobilization Processes for Heavy Metals in Calcium Silicate Hydrates. 2. Zinc. Langmuir.

[22] Ziegler F., Gieré R., Johnson C.A. (2001). Sorption Mechanisms of Zinc to Calcium Silicate Hydrate: Sorption and Microscopic Investigations. Environ. Sci. Technol..

[23] Stumm A., Garbev K., Beuchle G., Black L., Stemmermann P., Nüesch R. (2005). Incorporation of zinc into calcium silicate hydrates, Part I: Formation of C-S-H(I) with C/S=2/3 and its isochemical counterpart gyrolite. Cem. Concr. Res..

[24] Johnson C.A., Kersten M. (1999). Solubility of Zn(II) in Association with Calcium Silicate Hydrates in Alkaline Solutions. Environ. Sci. Technol..

[25] Ziegler F., Johnson C.A. (2001). The solubility of calcium zincate (CaZn_2_(OH)_6_.2H_2_O). Cem. Concr. Res..

[26] McWhitnney H.G., Cocke D.L. (1993). A surface study of the chemistry of zinc, cadmium and mercury in Portland cement. Waste Manag..

[27] Nochaiya T., Sekine Y., Choooun S., Chaipanich A. (2015). Microstructure, characterizations, functionality and compressive strength of cement-based materials using zinc oxide nanoparticles as an additive. J. Alloys Compd..

[28] Li X.G., Yin X.B., Ma B.G., Wu B., Chen Q., Lv Y. (2010). Investigation on Hydration Characteristics of Zinc-Doped Portland Cement Pastes. Adv. Mater. Res..

[29] Šiler P., Kolářová I., Novotný R., Másilko J., Pořízka J., Bednárek J., Švec J., Opravil T. (2017). Application of isothermal and isoperibolic calorimetry to assess the effect of zinc on cement hydration. J. Therm. Anal. Calorim..

[30] Coppola L., Coffetti D., Crotti E., Gazzaniga G., Pastore T. (2019). An Empathetic Added Sustainability Index (EASI) for Cementitious Based Construction Materials. J. Clean. Prod..

[31] Bougara A., Lynsdale C., Milestone N.B. (2018). The influence of slag properties, mix parameters and curing temperature on hydration and strength development of slag/cement blends. Constr. Build. Mater..

[32] Kledyński Z., Machowska A., Pacewska B., Wilińska I. (2017). Investigation of hydration products of fly ash–slag pastes. J. Therm. Anal. Calorim..

[33] Ogirigbo O.R., Black L. (2016). Influence of slag composition and temperature on the hydration and microstructure of slag blended cements. Constr. Build. Mater..

[34] Kalinkin A.M., Gurevich B.I., Myshenkov M.S., Kalinkina E.V., Zvereva I.A. (2018). A calorimetric study of hydration of magnesia-ferriferous slag mechanically activated in air and in CO2 atmosphere. J. Therm. Anal. Calorim..

[35] Castellano C., Bonavetti V., Donza H., Irassar E. (2016). The effect of w/b and temperature on the hydration and strength of blast furnace slag cements. Constr. Build. Mater..

[36] Šiler P., Bayer P., Sehnal T., Kolářová I., Opravil T., Šoukal F. (2015). Effects of high-temperature fly ash and fluidized bed combustion ash on the hydration of Portland cement. Constr. Build. Mater..

[37] Siler P., Kratky J., Kolarova I., Havlica J., Brandstetr J. (2013). Calorimetric determination of the effect of additives on cement hydration process. Chem. Pap..

[38] Siler P., Kratky J., De Belie N. (2012). Isothermal and solution calorimetry to assess the effect of superplasticizers and mineral admixtures on cement hydration. J. Therm. Anal. Calorim..

[39] Pacewska B., Wilińska I., Nowacka M. (2011). Studies on the influence of different fly ashes and Portland cement on early hydration of calcium aluminate cement. J. Therm. Anal. Calorim..

[40] Rahhal V., Cabrera O., Talero R., Delgado A. (2007). Calorimetry of Portland cement with silica fume and gypsum additions. J. Therm. Anal. Calorim..

[41] Brandštetr J., Polcer J., Krátký J., Holešinský R., Havlica J. (2001). Possibilities of the use of isoperibolic calorimetry for assessing the hydration behaviour of cementitious systems. Cem. Concr. Res..

[42] Shanahan N., Tran V., Zayed A. (2016). Heat of hydration prediction for blended cements. J. Therm. Anal. Calorim..

[43] European Committee for Standardization (2010). European Standard: Methods of Testing Cement—Part 9: Heat of Hydration—Semi-Adiabatic Method.

[44] Siler P., Kolarova I., Kratky J., Havlica J., Brandstetr J. (2014). Influence of superplasticizers on the course of Portland cement hydration. Chem. Pap..

[45] Ježo L., Palou M., Kozánková J., Ifka T. (2010). Determination of activation effect of Ca(OH)_2_ upon the hydration of BFS and related heat by isothermal calorimeter. J. Therm. Anal. Calorim..

[46] Šoukal F., Koplík J., Ptáček P., Opravil T., Havlica J., Palou M.T., Kalina L. (2016). The influence of pH buffers on hydration of hydraulic phases in system CaO–Al_2_O_3_. J. Therm. Anal. Calorim..

[47] Gruyaert E., Robeyst N., De Belie N. (2010). Study of the hydration of Portland cement blended with blast-furnace slag by calorimetry and thermogravimetry. J. Therm. Anal. Calorim..

[48] Haines P.J. (2002). Principles of Thermal Analysis and Calorimetry.

[49] Bensted J. (1987). Some applications of conduction calorimetry to cement hydration. Adv. Cem. Res..

[50] De Schutter G., Taerwe L. (1995). General hydration model for Portland cement and blast furnace slag cement. Cem. Concr. Res..

[51] De Schutter G. (1996). Fundamental and Practical Study of Thermal Stresses in Hardening Massive Concrete Elements. Ph.D. Thesis.

[52] Poppe A.-M., De Schutter G. (2005). Cement hydration in the presence of high filler contents. Cem. Concr. Res..

[53] Maciel M.H., Soares G.S., Romano R.C.D.O., Cincotto M.A. (2018). Monitoring of Portland cement chemical reaction and quantification of the hydrated products by XRD and TG in function of the stoppage hydration technique. J. Therm. Anal. Calorim..

[54] Novotný R., Bartoníčková E., Švec J., Mončeková M. (2016). Influence of active alumina on the hydration process of Portland cement. Procedia Eng..

[55] Da Silva R., Reis C.A.R.J.P., Lameiras F.S., Vasconcelos W.L. (2002). Carbonation-related microstructural changes in long-term durability concrete. Mater. Res..

